# Uganda’s increasing dependence on development partner’s support for immunization – a five year resource tracking study (2012 – 2016)

**DOI:** 10.1186/s12889-021-10178-0

**Published:** 2021-01-19

**Authors:** Carol Kamya, Christabel Abewe, Peter Waiswa, Gilbert Asiimwe, Faith Namugaya, Charles Opio, Immaculate Ampeire, Stephen Lagony, Charlotte Muheki

**Affiliations:** 1 Infectious Diseases Research Collaboration, Kampala, Uganda; 2 Health Net Consult, Kampala, Uganda; 3 Department of Health Policy,Planning and Management, School of Public Health, Makerere University, Kampala, Uganda; 4 Uganda & Global Health Division, Karolinska Institutet, Solna, Sweden; 5 Expanded Program on Immunisation, Ministry of Health, Kampala, Uganda

**Keywords:** Immunization, Sustainability, Resource tracking, Financial flows, Financing, Gavi, Uganda

## Abstract

**Background:**

In Uganda, there are persistent weaknesses in obtaining accurate, reliable and complete data on local and external investments in immunization to guide planning, financing, and resource mobilization. This study aimed to measure and describe the financial envelope for immunization from 2012 to 2016 and analyze expenditures at sub-national level.

**Methods:**

The Systems of Health Accounts (SHA) 2011 methodology was used to quantify and map the resource envelope for immunization. Data was collected at national and sub-national levels from public and external sources of immunization. Data were coded, categorized and disaggregated by expenditure on immunization activities using the SHA 2011.

**Results:**

Over the five-year period, funding for immunization increased fourfold from US$20.4 million in 2012 to US$ 85.6 million in 2016. The Ugandan government was the main contributor (55%) to immunization resources from 2012 to 2014 however, Gavi, the Vaccine Alliance contributed the majority (59%) of the resources to immunization in 2015 and 2016. Majority (66%) of the funds were managed by the National Medical Stores. Over the five-year period, 80% of the funds allocated to immunization activities were spent on facility based routine immunization (expenditure on human resources and outreaches). At sub-national level, districts allocated 15% of their total annual resources to immunization to support supervision of lower health facilities and distribution of vaccines. Health facilities spent 5.5% of their total annual resources on immunization to support outreaches.

**Conclusion:**

Development partner support has aided the improvement of vaccine coverage and increased access to vaccines however, there is an increasing dependence on this support for a critical national program raising sustainability concerns alongside other challenges like being off-budget and unpredictable. To ensure financial sustainability, there is need to operationalize the immunization fund, advocate and mobilize additional resources for immunization from the Government of Uganda and the private sector, increase the reliability of resources for immunization as well as leverage on health financing reforms like the National Health Insurance.

## Background

Immunization is a cost-effective intervention that has played a vital role in controlling and eliminating vaccine-preventable diseases. Globally, immunization is estimated to avert approximately 2–3 million deaths each year as access to immunization services has improved especially among the hard-to-reach and vulnerable populations [1]. Uganda is a low-income country with a population of 34.9 million people [2]. In line with the Sustainable Development Goal 3.2 that seeks to end preventable deaths of newborns and children under 5 years of age [3], the Uganda National Expanded Program on Immunization (UNEPI) has increased access to immunization services nationally through the introduction of new vaccines and improvement in vaccine coverage with support of its partners [4–6]. Coverage estimates show high performance for third dose of Diphtheria, Tetanus and Pertussis -DPT3 (93%), BCG (88%), Measles (87%), Polio 3 (92%) and Pneumococcal Conjugate (92%) vaccines [4].

The Global Vaccine Action Plan highlights the need to increase the total amount of funding for immunization from countries and development partners even though financing for immunization is primarily the responsibility of governments [7]. In order to ensure predictable and sustainable funding for immunisation, resource tracking efforts are needed to guide governments and partners [7]. Resource tracking in Uganda has been possible through the institutionalization of the National Health Accounts (NHA) that has provided evidence to monitor health financing since 1997 [8]. Findings from the NHA show that the Government of Uganda’s (GOU) expenditure on health for 2016/17 & 2017/18 fiscal years was US$1.8 billion representing 1.1% of the Gross Domestic Product [9]. However, Uganda’s budget allocated to health (8.7%) is below the Abuja declaration target of 15% [9, 10]. Additionally, the health expenditure per capita (US$56) is also lower than US$84 per capita recommended by the World Health Organization (WHO) [9]. The current health expenditure estimates also highlight an increase in public and private funds but a decrease in funding from development partner funds [9]. In addition to the NHA, WHO/UNICEF jointly capture various domains on performance, planning, financing and quality indicators from member states through the Joint Reporting Form (JRF) so as to track implementation of the Global Vaccine Action Plan (GVAP) [11]. From the JRF, the GOU’s spending on routine immunization per surviving infant has increased from $3 (2006) to $11 (2014) [12]. More recent estimates in 2017 show that the GOU contributes approximately 35% of the total expenditure on immunization [13]. Nevertheless, Uganda has not consistently reported on JRF indicators and therefore exacerbating the need for resource mapping exercises.

A mapping of financial flows for immunization in 2009 showed an increase in financing for routine immunization in Uganda from $24.2 million to $32.9 million in 2009/10 and 2010/11 respectively with the GOU contributing approximately half of all the resources [14]. There is a dearth of evidence in the financial flows specific to immunization especially in light of the newly introduced vaccines. Additionally, persistent weaknesses have been noted in obtaining accurate reliable and complete data on local and external investments in immunization and yet these are critical to estimate costs, resource needs and resource gaps. It is vital to have accurate data on funding flows and expenditure to aid country level planning, financing and also resource mobilization.

Gavi, the Vaccine Alliance (Gavi) commissioned a prospective evaluation in four countries including Uganda with an aim of generating evidence regarding the relevance, effectiveness, impact and efficiency of their support. A key evaluation question was centered on identifying how Gavi resources are utilized and their relationship with domestic and development partners resources. This paper presents findings from the resource tracking sub-study that traced the flow of immunization resources from national to sub-national levels. The objectives were: 1) to measure and describe the financial envelope for immunization activities at national level in Uganda from 2012 to 2016, and 2) to conduct an expenditure analysis of the resources received and utilized in 2015 and 2016 at sub-national level.

## Methods

### Overview of national health system in Uganda

The national health system in Uganda comprises of the private and public sector. The private sector includes private for profit and private not for profit providers while  the public sector constitutes all government health facilities under the Ministry of Health and the Ministries of Defense, Internal Affairs, Education and the Local Government [15]. Health services in Uganda are provided through a central and decentralized system at national level, districts and health sub-districts. At national level, services are delivered through national hospitals and regional hospitals while at district level, services are provided through general hospitals, Health Center (HC) IVs, HC IIIs, HC IIs and village health teams [16]. Hospitals provide technical back up for referral and support functions to district health services. The health sub-districts are housed at HCIV’s whose responsibility is to plan, organize, budget and manage health services at the facility but also responsible for lower health center levels [16]. HCIIIs provide preventive, promotive and curative care while HCIIs provide the first level of interaction between the health sector and communities [16].

### Study site and population

This study was conducted at both national and sub-national levels in Uganda. At national level, we studied immunization stakeholders from public entities, development partners and international non-governmental organizations. In order to capture the role of decentralization, we studied districts and health facilities. As such, District Health Offices (DHO), Chief Administrative Officers, health facility managers and Expanded Program on Immunization (EPI) focal persons at districts and health facilities were included in the study.

### Quantifying the resource envelope at national level

#### Approach

To quantify the total resource envelope for immunization, a resource mapping methodology was used. This approach maps both financial and non-financial (commodity and equipment) resources for immunization. As such, we identified initial key immunization stakeholders through document review and additional respondents were identified by key informants who had been interviewed. These stakeholders were broadly categorized as public entities, development partners, and international non-governmental organizations. Consequently, these were grouped into financing sources, financing agents and service providers in line with the System of Health Accounts (SHA) 2011 classifications [17]. The SHA codes for health care functions for immunization (HC.6.2) were further disaggregated to allow for greater detail on the types of immunization activities.

#### Scope

The scope of analysis included all public and external sources of financing or commodities in Uganda. Following the previous resource tracking efforts conducted in 2011, the study sought to collect data for subsequent financial years: 2011/12 (2012), 2012/13 (2013), 2014/15 (2014), 2015/16 (2015), and 2015/16 (2016). Data were collected in three phases: 2013, 2014 and 2016.

#### Estimation

To estimate the total envelope of immunization funds, we summed the a) total funds directly to support immunization (funds from development partners + GOU contribution at national level) and b) GOUs expenditure on salaried labor (% attributed to immunization) and proportion of Primary Health Care (PHC) funds spent on immunization at sub-national level. PHC funds in Uganda are part of the health sector grants provided to local governments and health facilities in order to facilitate the provision of health services with emphasis on access, quality and affordability [18]. These funds include wage conditional grants, non-wage conditional grants, transitional development-sanitation and transitional development [18]. PHC funds are released on a quarterly basis from the Ministry of Finance to district local governments (district health offices and hospitals) and to health facilities.

### Expenditure analysis at sub-national level

The main objective of the expenditure analysis at district level was to estimate and describe what immunization resources were received and how they were utilized over two financial years - 2015/16 (2015) and 2015/16 (2016). Data was collected from districts and health facilities.

#### District and health facility selection

Seven districts were included in the study. Districts were purposively selected based on the Reach Every District (RED) classification of districts which includes vaccine coverage performance [19] and geographic representation. Using this criterion, the following districts were sampled: Lamwo, Abim, Masindi, Mitooma, Nakaseke, Kween and Iganga. Data was collected from thirty-one sites (health facilities and district health offices). In each of the sampled districts, the District Health Office (*n* = 7) and three to four health facilities (*n* = 24) were studied. Health facilities were purposively selected based on their immunization performance/coverage of the third dose of Diphtheria, Pertussis Tetanus vaccine (DPT3), level of care (Hospital, Health Centers IV, III and II) and ownership (public and private-not-for-profit facilities).

#### Estimation of government of Uganda’s support at sub-national level

Estimation of the GOU's contribution can be under-estimated if expenditures on vaccines and operational costs is considered without the investment in human resources responsible for service delivery and other infrastructure. The estimation of the GOU's expenditure on human resources was outside the scope of this study. Nevertheless, the study used findings from the EPIC study to estimate the GOU's contribution to salaries for immunization (labor and Primary Health Care funds) and adjusted the estimates for inflation [20].

### Data collection and analysis

Being a retrospective quantitative study, this study relied on a combination of face-to-face key informant interviews, using structured data collection tools, and review of documents provided by respondents. Data was collected by research assistants trained on the SHA methodology and in the use of the data collection tools. Data was first captured using hard copies of the tools and then entered into pre-coded MS Excel® spreadsheets. Level one data cleaning and verification was conducted on data entered in the Excel spreadsheets. Thereafter, data were entered into an excel-based analysis screen and coded using the SHA (2011). Data were categorized according to stakeholder function (source, agent or provider) and further disaggregated into expenditure by program area as well as by immunization line items. Data was captured in Ugandan shillings and later converted to US dollars using an exchange rate of 1 US dollar to 3443 Ugandan shillings (US $1: UGX 3443).

## Results

### Overview of financing for immunization in Uganda

In Uganda, there are two financing schemes through which immunization funds are channeled: the government and “rest of the world” schemes. The government scheme represents public funds that are comprised of the GOU funds and on-budget funds from development partners. Financing agents for the public funds are Ministry of Health (MOH) / Uganda National Expanded Program on Immunization (UNEPI) and National Medical Stores (NMS). Providers of services for the public funds are: MOH / UNEPI, DHOs, government health facilities, and Private Not for Profit (PNFPs) health facilities. On the other hand, the “rest of the world” scheme is funded by development partners including United Nations agencies, bilateral agencies, and international Non-Government Organizations (NGOs). Development partners manage the bulk of their funds, with a few exceptions (e.g. World Health Organization and Gavi) whose bulk of the funds are managed by UNEPI and NMS (for vaccines, supplies procurement and handling). Service providers for development partner funds are UNEPI, DHOs, government health facilities, and non-government health facilities. In some cases, the development partners also provide services.

### Resource envelope for immunization

#### Financing sources

**Fig. 1:**
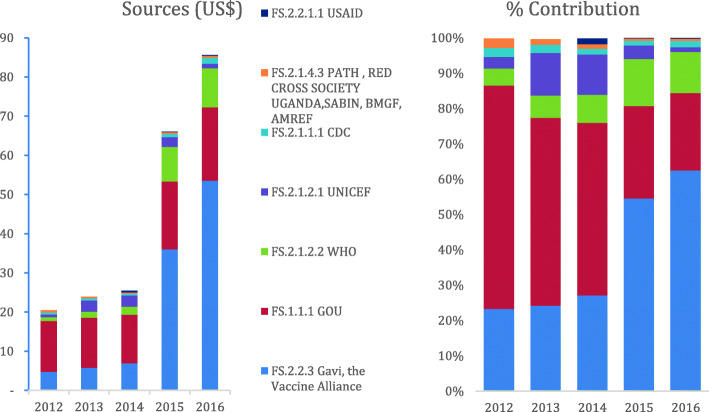
Sources of funding and their percentage contribution from 2012 to 2016

Over the five-year period, funding for immunization increased fourfold from US$20.4 million in 2012 to US$ 85.6 million in 2016. (Fig. 1). The main contributors to the resource envelope were GOU and Gavi. The contribution from the GOU steadily increased from US$12 million in 2012 to US$ 18 million in 2016 as such, the Ugandan government was the greatest contributor to the immunization resources (55%) from 2012 to 2014. However, in 2015 and 2016, Gavi contributed more resources accounting for 59% of the resource envelope. Other sources of funding over the five-year period included WHO (9%), UNICEF (6%), CDC (2%) and other partners (1%).

#### Financing agents

**Fig. 2:**
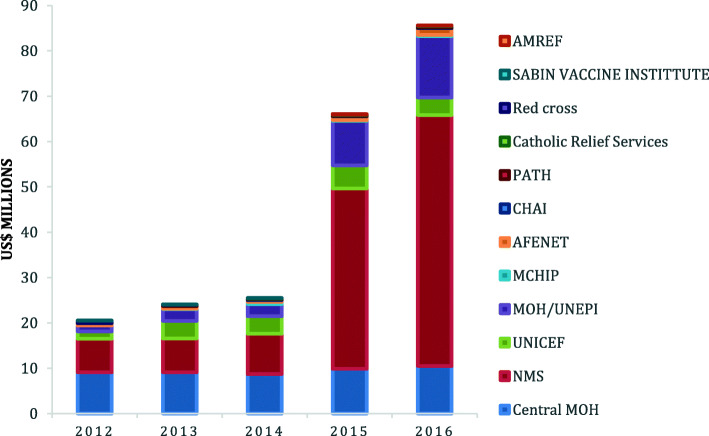
Trends in financing agents for Immunization funds in Uganda from 2012 to 2016

Notably, these immunization funds were managed by several public and non-public stakeholders including NMS, Ministry of Health, UNICEF, African Field Epidemiology Network (AFENET), PATH, Maternal and Child Health Integrated Program (MCHIP), Africa Medical Research Foundation (AMREF) Uganda, Catholic Relief Services, SABIN Vaccine Institute and Clinton Health Access Initiative (CHAI) (Fig. 2). NMS has progressively managed more immunization funds from 36% (US$ 7 million) in 2012 to about 66% (US$ 52 million) in 2016. Similarly, UNEPI managed more funds from 6% (US$ 1, million) in 2012 to 17% (US$13 million) in 2016.

#### Providers of immunization services

Providers of immunization services included government owned facilities at different levels including hospitals, DHOs, NMS, UNEPI and the multinational agencies. Government health facilities provided 70% (US$ 31million) of the immunization services over the five- year period. District Health Offices, administrative agencies (NMS and UNEPI) and developmental partners provided 15, 12 and 3% of the immunization services respectively.

#### Expenditure on immunization activities/health care function

Immunization resources were further categorized by health care function that is to say immunization activities/programs. Over the five-year period, 80% of the funding was spent on facility-based routine immunization which includes expenditure on human resources and outreaches (Table 1). The second largest expenditure (14%) was on immunization programs which includes expenditure on supplemental immunization activities, family health days and research. Immunization surveillance and activities that could not be disaggregated (expenditure on health system strengthening grant and new vaccine Introduction) took up 2%. Other immunization activities like training, social mobilization and advocacy only took up the rest of the expenditures over the study period.

**Table 1:** Expenditure of the resource envelope by immunization activity from 2012 to 2016

Immunization activity	Expenditure in US$				
	2012	2013	2014	2015	2016
Routine immunization (Facility based)	18,210,863	17,659,018	18,762,707	52,860,877	70,113,273
Immunization programs	929,422	4,676,155	4,821,377	10,920,709	9,613,709
Program management	58,089	29,044	261,400	203,311	290,444
EPI surveillance	377,578	232,356	580,889	464,711	1,336,044
Training	377,578	377,578	435,667	232,356	726,111
Not disaggregated	–	987,511	435,667	842,289	3,456,288
Supervision	–	–	145,222	290,444	
Social Mobilization and advocacy	464,711	87,133	29,044	232,356	145,222
	20,418,241	24,048,795	25,471,973	66,047,053	85,681,091

### Expenditure analysis at district and health facility levels

The study conducted an expenditure analysis to quantify and describe the immunization resources received and utilized at district level and health facility levels for financial years − 2015/16 (2015) and 2015/16 (2016). The expenditure analysis presents expenditures for immunization activities at district and health facility level both by program area and by line item classification.

### Expenditure analysis at district level

At district level, immunization activities were funded by the GOU through the PHC non-wage fund and development partners.

Table 2. Over the two-year period, total funds received by districts varied. WHO (57%) and UNICEF (23%) provided the largest proportion of funding in the selected districts. Government funding through PHC grant accounted for about 3% of the total resource envelope in the sampled districts.

**Table 2:** Sources of funding for immunization at district level

District	Ugandan Government (PHC) (US$)		Gavi, the Vaccine Alliance (US$)		WHO (US$)		UNICEF (US$)		Other (AFENET) (US$)	
	2015	2016	2015	2016	2015	2016	2015	2016	2015	2016
Abim	960	960	–	3726	–	37,954	11,266	–	–	–
Iganga	1191	1191	11,418	18,335	29,671	58,474	40,513	66,617	–	–
Kween	1949	1227	4834	14,285	53,991	54,202	2109	4650	–	–
Lamwo	–	–	–	7443	29,820	59,661	–	7931	474	7886
Masindi	1917	1685	8246	19,262	–	13,814	7570	7178	–	–
Mitooma	1296	1481	4990	16,512	24,451	37,772	–	6596	–	–
Nakaseke	1874	2007	5340	–	–	–	–	5799	–	–
Total	9187	8551	34,828	79,563	137,933	261,877	61,458	98,771	474	7886

#### Expenditure of public funds (Primary Health Care Grant) at district level

**Fig. 3:**
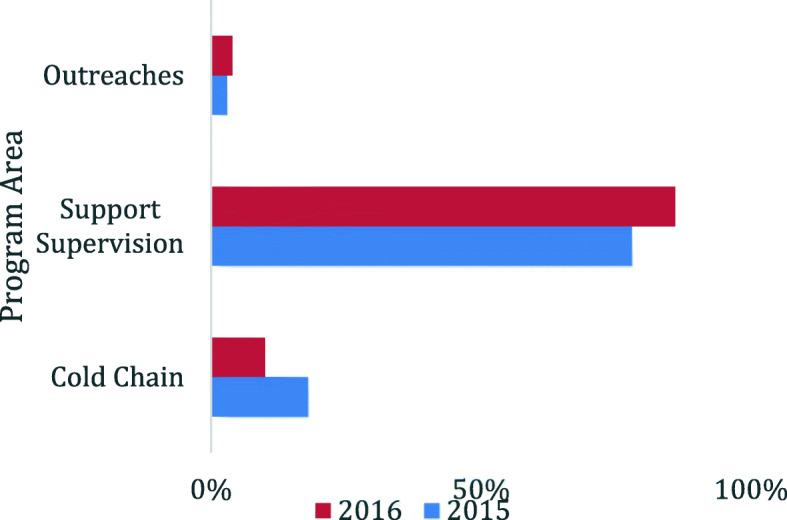
District expenditure of public funds on immunization by program area

Public funds (PHC grant) from the Ugandan government allocated to immunization specific activities varied by district. On average, district health offices spent 15% of their total annual resources on immunization activities. More than half of the districts (4 out of 7 districts) allocated less than 15% of their total funds (proportion of their PHC grant) to support immunization activities. The proportion of funds allocated to immunization ranged from 0 to 45% over the 2 years. By program area (Fig. 3), the bulk of funds were spent on supervision accounting for 78% in 2015 and 86% in 2016. Cold chain maintenance took up 18% in 2015 and 10% in 2016. The rest of the funds were spent on outreaches. By line item, the bulk (33%) of funds were spent on fuel for vehicles to transport health workers for outreaches and distribution of vaccines. Per-diems/allowances for outreaches took up 20 and 15% of the total PHC funds in 2015 and 2016. Activities relating to supervision and cold chain maintenance ranged between 15 and 19% in both 2015 and 2016.

#### Expenditure of funds from development partners at district level

**Fig. 4:**
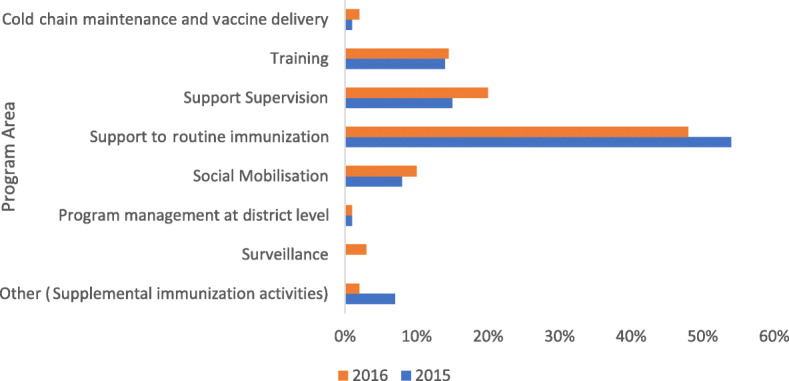
District Expenditure of funds from development partners by program area

Funds from development partners were mainly spent on routine immunization accounting for 51% over the study period (Fig. 4). Expenditure on routine immunization includes spending on vaccine collection and per-diems/allowances to support outreaches. Other expenditures included supervision to lower health centers, training, social mobilization, supplemental immunization activities, surveillance and cold chain maintenance and program management.

### Expenditure analysis at health facility level

The study further analyzed expenditure at health facility level to determine what proportion of the annual public funds were spent on immunization activities and what the expenditure drivers were.

In the districts visited, the annual average expenditure for immunization across all levels of care was 5% in 2015 and 6% in 2016 (Table 3). Hospitals spent 5 and 6% of their total immunization budget on immunization related activities as a proportion of average annual PHC funds received in 2015 and 2016 respectively. On average, HCIV’s, HCIIIs and HCIIs spent 26, 13.5 and 44% on immunization related activities respectively. Furthermore, the total PHC expenditure for each level of health facility was estimated according to program area. Outreaches consumed the bulk of immunization resources accounting for 87 and 88% in 2015 and 2016 respectively. Social mobilization and collection of vaccines separately accounted for 7% in 2015 and 6% in 2016 of the PHC funds. By line item, majority of the funds were spent on per-diems/allowances accounting for 67% in 2015 and 78% in 2016. This was followed by transport and fuel expenditures that accounted for 21 and 25% in 2015 and 2016 respectively. The remaining 12% in 2015 and 14% in 2016 was spent on social mobilization activities, cold chain maintenance and facilitation of vaccinators.

**Table 3:** Health facility average annual expenditure on immunization

Facility type	Average annual PHC US$	Average annual immunization expenditure US$	Average annual PHC US$	Average annual immunization expenditure US$
	2015	2016	2015	2016
Health Centre II (*N* = 7)	633.65	280.65	746.18	375.44
Health Centre III (*N* = 8)	4566.52	625.49	4752.09	613.46
Health Centre IV (*N* = 6)	4130.29	649.54	9851.28	1021.21
Hospital (*N* = 3)	41,279.29	1019.27	40,685.70	1592.95
Overall average	50,609.76	2574.95	56,035.24	3603.05

## Discussion

In Uganda, the resource envelope for immunization increased fourfold to US$ 85.6 million in 2016. The contribution from the Ugandan Government increased by US$ 5.7 million over the study period accounting for 55% of the resource envelope in 2012 to 2014. Despite the increase in its contribution for immunization, the Ugandan Government’s contribution to the resource envelope reduced to 24% in 2015 and 2016 as Gavi contributed more resources for immunization accounting for 59% of the resource envelope. Other development partners contributed 18% to the resource envelope. There is a substantial contribution (76%) of development partners in supporting the immunization program in Uganda. Similar resource tracking efforts estimate the government’s contribution to be 50% [14] indicating a reduction in its contribution to the resource envelope for immunization. Analyses from country multiyear plans (cMYPs) also estimate the government contribution to account for about 54% of the total routine program financing and non-vaccine financing respectively [21]. The increase in the resource envelope for immunization can partly be explained by the lift in the ban on Gavi funding to Uganda in 2012 following the misuse of funds [22, 23]. This ban was lifted following the institutionalization of fiduciary risk management approaches [24, 25]. Additionally, the introduction of new vaccines and implementation of other Gavi support can also explain the increase in the resource envelope for immunisation. Uganda introduced several new vaccines in quick succession; including Pneumococcal Conjugate Vaccine (PCV) in 2013, Human Papilloma virus vaccine (HPV) in 2015, Meningitis A in 2016 and Injectable Polio Vaccine (IPV) in 2016 [26]. In addition, the EPI conducted Meningitis A vaccine campaigns, shifted from the trivalent to the bivalent polio vaccine while implementing the first Health Systems Strengthening (HSSI) grant from Gavi during the study period [27].

The expansion of immunization schedules through introduction of new vaccines increases program and system costs of national immunization programs driven by high prices of new vaccines and program budgets have been shown to double or triple in countries graduating from Gavi support [21, 28, 29]. The increase in costs and resource requirements during and after new vaccine introduction is expected to be commensurate with increases in government expenditure on immunization, however government budgets may not easily absorb the portfolio of vital vaccines financed by Gavi [30, 31]. The Ugandan Government’s contribution to the resource envelope for immunization increased over the study period however, the magnitude of support from development partners raises sustainability concerns of a critical national program. Several low income countries also rely on the significant support from Gavi to meet the increasing needs of EPI programs to introduce and scale up vaccines however, there have been limited signs of the ability to transition to other sources of financing outside Gavi support with evident financing gaps to sustain the current immunization gains [32]. In Uganda, the sustainability concerns are exacerbated by a 90% (US$ 487.5 million) financial gap in the immunization resources required for Uganda over a five-year period (2016–2020) when Gavi’s contribution is excluded [33].

The achievement of sustainable immunization however, needs to be considered in the context of the broader health system financing landscape. Financing for health in Uganda is largely inadequate and has decreased overtime to 6.9% in 2015/16 [9, 34]. As such, the available financing is not sustainable to maintain high coverage rates and is exacerbated by new vaccine introduction. Inadequate financing for immunization coupled with the heavy reliance on development partner support also highlights challenges of relying on external funding as the support is often ‘off-budget’ making it difficult to plan and track expenditures, unpredictable due to the difficulty of making multiyear commitments and is often unevenly targeted in terms of its developmental impact mainly focused on financing recurrent costs (vaccines and supplies) rather than long term improvements [15, 28, 35].

Development partner support is expected to increase allocation of developing country resources towards health programs/immunization or even result into the same degree of benefit however, this cannot be guaranteed due to several complexities of the ability and willingness of governments to pay for health care [32, 36–38]. Gavi has not only increased access to lifesaving/underutilized vaccines and improved immunization coverage for new vaccines but, has also prioritized financial sustainability at country level despite the challenges and limitations in its approaches [32, 39–42]. Financial sustainability has been prioritized through its policies on eligibility, transition and financing to enable countries fully finance immunization programs beyond the time limited contribution from Gavi [28, 32, 43, 44]. Alongside Gavi’s initiatives to ensure sustainability at country level, EPI programs need to prioritize financial sustainability planning for immunization as it has been shown to have a positive impact on mobilization of resources [45]. Closing funding gaps for immunization and achieving financial sustainability requires a significant increase in the public sector budget, increase in government commitments for immunization, greater commitments from development partners and a reduction in vaccine prices in the context of Gavi funding so as to allow countries to transition [32, 45, 46]. In Uganda, an immunization bill was passed and enacted into law in 2016 and has the provision for the establishment of an immunization fund to facilitate mobilization of domestic resources for immunization however, it is not yet operational [26]. These funds are meant to support purchasing of vaccines and related supplies, cold chain as well as other immunization activities.

Findings on the expenditure by immunization activity showed that the majority of the funds are spent on facility based routine immunization. Over the study period, there was a 285% increase in the expenditure on facility based routine immunization mainly driven by spending on human resources which includes salaries for health workers. Facility based routine costs have been shown to take up the majority of the immunization expenditure driven by expenditure on human resources as it is a key driver of program resources after the expenditure on vaccines and supplies [32, 47]. Our findings also highlighted a change in management of funds at national level with National Medical Stores managing more funds in 2016. This change can be attributed to the organizational changes in which the responsibility for supplies vaccine logistics management, vaccine quality and safety were shifted from UNEPI to National Medical Stores in April 2012 [48]. As such this meant less financial flow of funds to UNEPI. However, due to the Health System Strengthening grant and several new vaccine introductions, UNEPI is also managing more funds (17%) despite the organizational changes when compared to the 8% it was managing in 2010 [47].

At district level, the district health office received immunization funding from the government (PHC grant) and development partners. WHO provided the largest proportion (57%) of the funds in the districts visited. More than half of the districts allocated less than 15% of the total annual resources (PHC grant) to immunization activities which translates to US$ 1452 annually per district. The PHC grant received on a quarterly basis to support all health programs as well as facilitate facility supervisions, vaccine delivery, cold chain maintenance and meetings is inadequate [18]. Due to the decentralized system in Uganda, the allocation of the PHC grant to immunization varies by district and is highly dependent on the degree of prioritization of immunization activities and availability of additional resources from development partners.

At health facility level, the overall annual average expenditure on immunization (PHC grant) across all levels of care was 5.5% over the two-year period. Majority of the facilities spent their PHC grant on outreaches (88%) specifically on per-diems/ allowances for staff. These findings show an increase in resources allocated to outreaches when compared to an earlier costing analysis that showed that health facilities were allocating only 28% of their resources to outreaches [47]. Majority of services are being provided by government health facilities of which the bulk of the funding supports facility based routine immunization activities. These findings are consistent with a similar study that also showed that the largest proportion of funding was devoted to routine facility based immunization with an average of 40% across all levels of care [14]. Therefore, majority of the funds are still spent at the level of service delivery.

This study had limitations but, they are unlikely to affect the findings and conclusions. The district level expenditure analyses only purposively sampled seven districts due to budget constraints and therefore findings cannot be generalized to all districts. Despite this, the study ensured that the district selection accounted for performance in line with RED strategy, geographically representation and vaccine coverage performance. Also, the study triangulated findings at national, district and health facility levels. Further, the Government of Uganda resources at sub-national/district level could have been underestimated given that we did not include the cost of salaried labor, purchase, storage, and distribution of vaccines. Despite this, this contribution was accounted for at national level using previous costing estimates [47].

## Conclusions

The resource envelope for immunization has increased fourfold since 2012 to a total of US$ 85.6 million in 2016 and is mainly attributed to new vaccine introduction and the lift of the ban of Gavi funding to Uganda in 2012. The Ugandan Government was the greatest contributor of immunization resources but Gavi, the Vaccine Alliance is now the greatest contributor of the immunization envelope in Uganda. Districts allocated 15% of their total annual resources to immunization to support supervision of lower health centers and distribute vaccines. Health facilities spent about 5.5% of their total annual resources to support outreaches. Although the support from development partners has facilitated the improvement of immunization coverage and new vaccine introduction in Uganda, with Gavi and other development partners contributing the bulk of funding for immunization, it raises sustainability concerns of a critical national program. Additionally, development partners support is often off-budget and unpredictable. The immunization financing in Uganda is not sustainable to maintain high coverage rates and this is exacerbated by high costs of newly introduced vaccines. To ensure financial sustainability of the immunization program, findings from this study emphasize the need to operationalize the immunization fund in the immunization act, advocate and mobilize additional resources for immunization from the ministry of health and private sector, increase the reliability of resources for immunization as well as leverage on health financing reforms like the National Health Insurance. Additionally, on budget funding from development partners would guide national programs plan adequately. At district and health facility level, there is need to ring-fence resources for immunization. This study calls for continuous tracking of resources for immunization.

## Data Availability

The datasets used and/or analyzed during the current study are no publicly available but may be made available from the corresponding author on reasonable request.

## Abbreviations

BCG: Bacillus Calmette-Guerin
cMYP: Country Multi Year Plans
DHO: District Health Officer
EPI: Expanded Program on Immunization
Gavi: Gavi, the Vaccine Alliance
GOU: Government of Uganda
HC: Health Center
MOH: Ministry of Health
HSS: Health Systems Strengthening
NGOs: Non-Government Organizations
NHA: National Health Accounts
NMS: National Medical Stores
PHC: Primary Health Care Grant
RED: Reach Every District
SHA: System of Health Accounts
UNEPI: Uganda National Expanded Program on Immunization
